# The Double Burden of Malnutrition in Bangladesh: Policy Architecture, Implementation Realities, and Future Directions

**DOI:** 10.1016/j.cdnut.2026.109433

**Published:** 2026-07-25

**Authors:** Ashis Talukder, Matthew Kelly, Sanjoy Kumar Chanda, Darren Gray, Haribondhu Sarma

**Affiliations:** 1 National Centre for Epidemiology and Population Health, College of Health and Medicine, The Australian National University, Canberra, ACT, Australia; 2 Statistics Discipline, Science Engineering and Technology School, Khulna University, Khulna, 9208, Bangladesh; 3 Sociology Discipline, Social Science School, Khulna University, Khulna, 9208, Bangladesh; 4 Population Health Department, QIMR Berghofer Medical Research Institute, Brisbane, QLD, Australia

**Keywords:** double burden of malnutrition, nutrition policy, double-duty actions, Bangladesh, qualitative research

## Abstract

**Background:**

Bangladesh faces a growing double burden of malnutrition (DBM), where persistent undernutrition coexists with rapidly rising overweight and obesity. Although the country has a comprehensive National Nutrition Policy architecture, it remains unclear whether current policies address DBM as an integrated challenge.

**Objectives:**

This study aimed to explore stakeholders’ perspectives on the DBM in Bangladesh by examining how DBM is understood and framed, assessing the responsiveness of the current national nutrition policy architecture, identifying implementation challenges, and exploring policy and governance priorities for strengthening future nutrition outcomes.

**Methods:**

This study employed a qualitative design conducted in Bangladesh between June and August 2025. A total of 12 purposively selected senior-level nutrition stakeholders participated in semistructured key informant interviews (KII), including representatives from government, international and national nongovernmental organizations, and research institutions. Participants were selected on the basis of their expertise and direct involvement in nutrition policy development and implementation. Interview data were analyzed using reflexive thematic analysis following Braun and Clarke, with themes generated inductively from participants’ accounts. The integrated Health Policy Triangle–Centers for Disease Control and Prevention Policy Process Framework was used as an interpretive lens to contextualize and organize the emergent themes within established health policy frameworks.

**Results:**

KII generated 4 themes that extend beyond policy description to illuminate implementation realities, causal explanations, and reform priorities: *1*) growing recognition of DBM among senior stakeholders that has not yet translated to frontline or community levels; *2*) stakeholder-confirmed undernutrition-focused policy architecture, with explanatory accounts of the cognitive and institutional barriers preventing DBM integration; *3*) implementation challenges characterized by symbolic multisectoral coordination, weak accountability systems without DBM-specific indicators, and limited subnational workforce capacity; and *4*) stakeholder-identified priorities including stronger multisectoral governance, food system regulatory reform, DBM-focused capacity building, and adaptation of existing programs as double-duty actions.

**Conclusions:**

Recognition of DBM in policy is necessary but insufficient without broader reforms in governance, program design, and resource allocation. The ongoing revision of Bangladesh’s Third National Plan of Action for Nutrition provides a timely opportunity to incorporate operational DBM indicators, adapt existing programs as double-duty actions, and strengthen accountability systems to track progress across multiple forms of malnutrition. These findings may also inform policy responses in other low- and middle-income countries undergoing similar nutrition transitions.

## Introduction

In recent decades, low- and middle-income countries (LMICs) have confronted an increasingly complex nutrition challenge: the simultaneous coexistence of undernutrition and overnutrition within the same populations, households, and even individuals across the life course, a phenomenon known as the double burden of malnutrition (DBM) [1,2]. Driven by rapid economic development, urbanization, and shifts in dietary patterns, this transition has progressed rapidly in many countries across Asia and Africa, placing mounting pressure on policy systems designed primarily for single-burden responses [3,4]. Traditional policy approaches that address undernutrition and overnutrition separately are increasingly inadequate because both forms of malnutrition share common upstream determinants, including poverty, food insecurity, poor dietary quality, and inequitable food environments [5]. Moreover, the consequences of early-life undernutrition can increase the risk of obesity and diet-related noncommunicable diseases later in life, highlighting the need for coordinated, life-course policy responses [6]. Fragmented policy frameworks may therefore generate unintended consequences, such as nutrition programs that increase caloric intake without improving diet quality, potentially accelerating the transition toward overnutrition [[7], [8], [9]].

The presence of DBM is particularly evident across LMICs. Globally, >45 countries experience both high levels of child stunting (>20%) and adult overweight (>35%), illustrating the extensive and simultaneous presence of undernutrition and overnutrition within populations [10]. In South Asia, the proportion of households affected by both forms of malnutrition ranges from 6% to 20%, demonstrating that DBM occurs not only at the population level but also within households [11]. In response to this growing challenge, global health agencies increasingly advocate for integrated actions that address the shared determinants of undernutrition and overnutrition simultaneously. These include promoting breastfeeding, improving maternal and child nutrition during the first 1000 d of life, creating healthier school food environments, and regulating the marketing of unhealthy foods to children. Such interventions have the potential to reduce undernutrition while also preventing overweight, obesity, and diet-related noncommunicable diseases [1,12,13].

Bangladesh provides a compelling example of DBM during a rapid nutrition transition. Despite substantial progress in reducing child undernutrition over the past 2 decades, undernutrition remains a major public health challenge. According to the most recent Bangladesh Demographic and Health Survey 2022, 22.5% of children aged <5 y were stunted and 9.7% were wasted, with the prevalence of wasting remaining nearly double the global target of <5% [[14], [15], [16]]. Micronutrient deficiencies also remain widespread, with anemia affecting children, adolescent girls, and women of reproductive age, and deficiencies in vitamin A, iron, iodine, zinc, vitamin B12, and folate reported among young children, pregnant women, and lactating mothers [17]. These forms of undernutrition are concentrated among young children, adolescent girls, and poorer rural populations [18,19]. At the same time, overweight and obesity have risen sharply, with 36.5% of women aged 15 to 49 y classified as overweight or obese in 2022, compared with only 9% in 2004 [14,20]. This increase has been most pronounced among older, wealthier, and urban women, contributing to growing socioeconomic and rural–urban inequalities in nutritional outcomes [21]. The simultaneous presence of persistent undernutrition and micronutrient deficiencies alongside rapidly increasing overweight and obesity illustrates the complex nature of malnutrition in Bangladesh and highlights the need for integrated policy responses that address all forms of malnutrition simultaneously [22].

Bangladesh’s nutrition policy architecture is guided by several key policy instruments. The National Nutrition Policy (NNP) (2015) [23] provides the overarching strategic framework, whereas the Second National Plan of Action for Nutrition (NPAN-2) [17] translates this framework into action through a multisectoral implementation strategy involving 17 ministries across areas such as life-course nutrition, agriculture and dietary diversification, and social protection. The Bangladesh National Nutrition Council (BNNC) is responsible for coordinating nutrition-related activities across 22 ministries [17,22]. NPAN-2 established specific targets for reducing undernutrition and overnutrition, including reducing stunting among children aged <5 y to 25% and limiting overweight and obesity among women of reproductive age to 30% by 2025 [17]. Although the stunting target has been achieved, the target for overweight and obesity has not been met. Complementing these efforts, the National Food and Nutrition Security Policy (2020) and its associated Plan of Action (2021–2030) [24] adopt a broader food systems approach aimed at improving dietary quality and promoting dietary diversification. As part of this strategy, the policy seeks to reduce the contribution of cereals to total dietary energy intake to <60% by 2030 [24].

Despite this comprehensive policy architecture, implementation priorities have historically remained focused on reducing undernutrition, particularly among children and women, with comparatively limited operational attention to overnutrition [22]. Although global evidence increasingly promotes integrated “double duty” actions that simultaneously address undernutrition and overnutrition [5], it remains unclear to what extent such approaches are operationalized within national implementation in Bangladesh. In addition, there is limited empirical evidence on how key stakeholders interpret and translate these policies in practice. To the best of our knowledge, no previous study has systematically examined how policymakers, program implementers, and technical experts understand DBM, assess existing policy responses, identify implementation barriers, and propose future policy directions in Bangladesh. Addressing this gap is essential for developing more coherent and effective strategies to strengthen nutrition policy implementation and tackle the growing prevalence of DBM.

This study addresses these gaps by investigating the perspectives of senior-level key stakeholders on DBM policy in Bangladesh. This study specifically explores the following: *1*) how stakeholders understand and frame DBM within Bangladesh’s nutrition transition context; *2*) how stakeholders perceive and assess current NNP architecture in relation to DBM; *3*) what implementation challenges constrain the translation of policy commitments into effective action; and *4*) which policy and governance strategies stakeholders consider most important for improving future nutrition outcomes. By answering these questions, we aim to generate stakeholder-grounded evidence to inform the strengthening of nutrition governance and integrated policy responses to DBM in Bangladesh.

## Methods

### Study setting

This study was conducted in Bangladesh between June and August 2025, coinciding with the final implementation phase of NPAN-2 and the preparatory period for its successor Third National Plan of Action for Nutrition (NPAN-3). As an LMIC experiencing DBM and undertaking a revision of its national nutrition plan, Bangladesh provided a timely context for capturing stakeholder perspectives on current nutrition policy challenges and priorities for future reform.

### Conceptual framework

**FIGURE 1:**
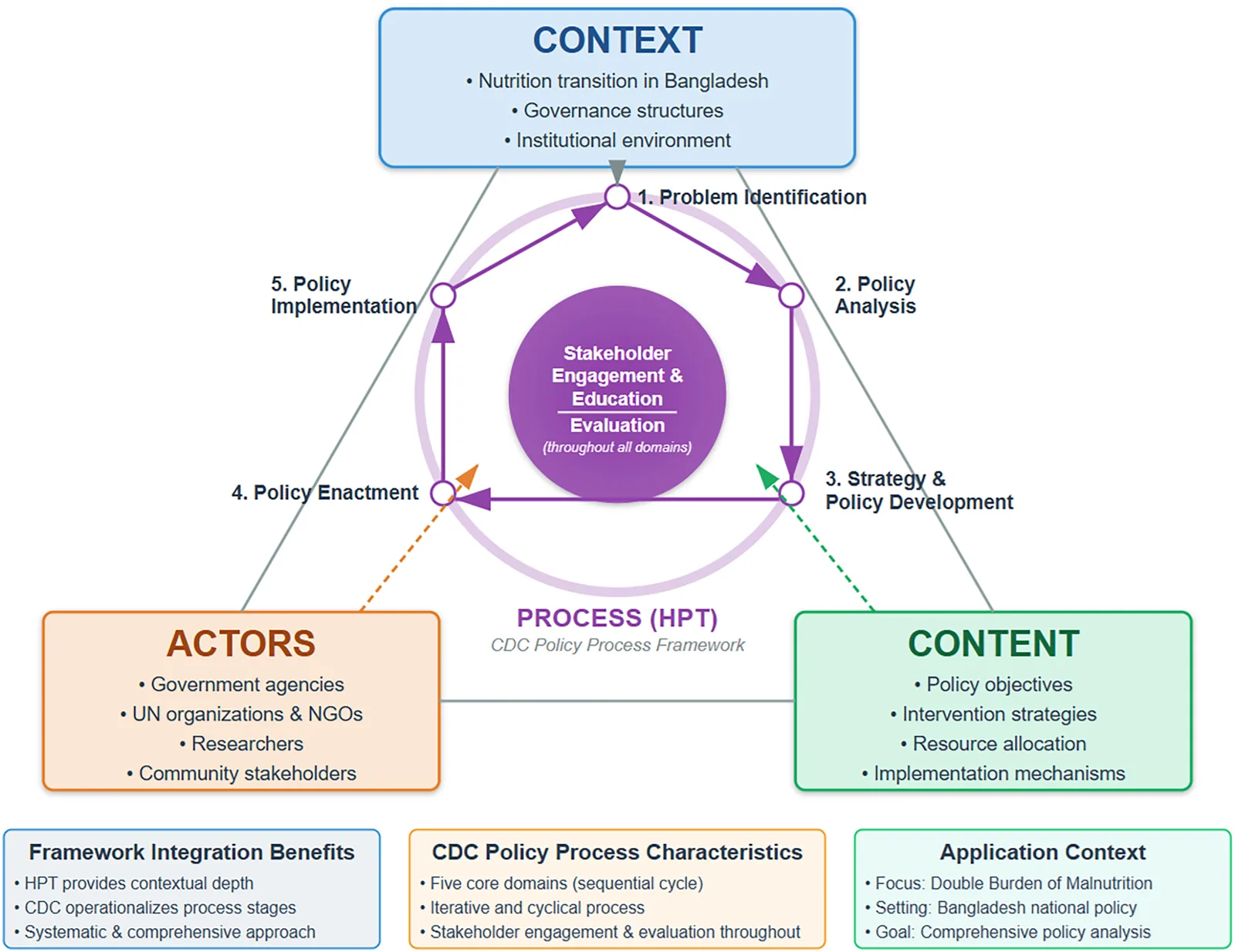
Integrated conceptual framework combining the Health Policy Triangle (HPT) and CDC Policy Process Framework for DBM policy analysis in Bangladesh. Note: The triangle represents the 3 HPT dimensions (context, actors, and content) that interact with the central process component, operationalized through 5 CDC framework core domains. Arrows indicate dynamic interactions between components. The framework acknowledges that policy processes are iterative and cyclical, continuously shaped by contextual factors, actor dynamics, and policy content. CDC, Centers for Disease Control and Prevention; DBM, double burden of malnutrition; NGO, nongovernmental organization.

This study adopted an integrated conceptual framework combining Walt and Gilson’s [25] Health Policy Triangle (HPT) with the Centers for Disease Control and Prevention (CDC) Policy Process Framework (HPT–CDC) [26] (Figure 1). The HPT examines how context (Bangladesh’s nutrition transition, governance structures, and institutional environment), actors [government agencies, United Nations (UN) organizations, nongovernmental organizations (NGOs), researchers, and community stakeholders], policy content, and policy processes interact to shape national DBM responses. The CDC Policy Process Framework operationalizes the HPT’s “process” component through 5 core domains: *1*) problem identification, *2*) policy analysis, *3*) strategy and policy development, *4*) policy enactment, and *5*) policy implementation, with 2 overarching domains, such as stakeholder engagement and education, and evaluation, operating continuously throughout the process.

This integration addresses each framework’s limitations. The HPT provides contextual depth but lacks detailed process guidance, whereas the CDC framework offers clear process delineation but limited attention to contextual and actor-related factors [26,27]. Together, the frameworks enable systematic examination of both structural determinants (context, actors, and content) and dynamic processes (from problem identification to implementation) that shape DBM policy responses. This integrated approach is particularly relevant for DBM, which requires coordinated multisectoral action, engagement of diverse stakeholders with competing priorities, and navigation of complex contextual factors, including nutrition transition, institutional legacies, and resource constraints.

### Participant selection and recruitment for key informant interviews

Participants were selected using purposive sampling to obtain a range of perspectives from stakeholders involved in nutrition policy and programming in Bangladesh. Although effective responses to DBM require coordinated action across multiple sectors, this study focused specifically on stakeholders working within the nutrition sector because its primary aim was to examine how DBM is understood, prioritized, and addressed within Bangladesh’s nutrition policy and program architecture. The implications of not including stakeholders from other sectors are discussed in the “study limitations” section.

Eligible participants had the following: *1*) current or recent involvement in nutrition policy development, program implementation, or technical advisory roles; *2*) ≥5 y of experience working on nutrition issues in Bangladesh; and *3*) the ability to provide informed perspectives on policy responses to malnutrition. To ensure variation across key stakeholder groups within the nutrition sector, participants were recruited from 4 categories: government nutrition authorities, international NGOs, national research and academic institutions, and subnational health and nutrition service providers. This approach enabled the inclusion of perspectives from individuals involved in policy development, technical advice, program coordination, and frontline implementation at both national and subnational levels. Initial participants were identified through a review of nutrition policy documents, national coordination meeting records, and relevant literature, with additional participants recruited through snowball sampling [28,29]. Potential participants were contacted by e-mail and provided with study information before providing informed consent. Individuals who were approached but declined to participate or did not respond were not formally logged. All individuals who agreed to participate completed an interview.

### Data collection

A semistructured interview guide was developed to explore participants’ perceptions and experiences of DBM policy in Bangladesh. The guide was initially developed by the first author (AT) following a review of relevant literature and informed by the integrated HPT–CDC conceptual framework (Figure 1). It was further refined through iterative discussions within the research team to ensure conceptual alignment with study objectives, clarity of wording, and logical sequencing of questions. Attention was given to ensuring that questions were clearly structured and that probes were appropriate to elicit detailed and in-depth responses from participants. The final version of the interview guide is provided in Supplementary Table 1 (File 1).

The interview guide included open-ended questions organized into 4 main domains: *1*) participants’ awareness and perceptions of DBM within the context of Bangladesh’s nutrition transition; *2*) perspectives on existing NNPs, including their scope in addressing undernutrition and overnutrition, and perceived policy gaps; *3*) stakeholder roles, coordination mechanisms, and multisectoral governance arrangements; and 4) experiences of policy implementation, monitoring and evaluation processes, and views on future policy directions. The use of open-ended questions allowed participants to introduce additional issues not anticipated a priori, in line with the flexible and exploratory nature of semistructured interviews. The guide was designed to ensure comprehensive coverage of relevant policy domains while maintaining consistency across interviews.

Face-to-face interviews were conducted by the first author (AT) between June and August 2025. AT had prior experience conducting research on nutrition and health-related topics in Bangladesh. Before data collection commenced, AT received training and mentorship in qualitative interviewing techniques, including probing, active listening, reflexive note-taking, and management of interviewer bias, from the senior author (HS), who has extensive experience in qualitative health policy research in low- and middle-income settings. HS also provided ongoing methodological oversight throughout the study. AT was not affiliated with any of the participating institutions and had no prior professional, supervisory, or working relationships with the participants. This independent position helped minimize the potential influence of existing organizational relationships, professional hierarchies, or perceived conflicts of interest on participant responses. After the first 2 interviews, HS reviewed the transcripts and field notes and provided structured feedback on question framing and probing depth, which informed minor refinements to the interviewing approach for subsequent interviews. Reporting of the qualitative methods follows the Consolidated Criteria for Reporting Qualitative Research checklist [30], provided in Supplementary Table 2 (File 2). All participants, including those representing international organizations, were Bangladeshi nationals based in Dhaka and fluent in Bangla. Consequently, all interviews were conducted in Bangla. Only the participant and the interviewer (AT) were present during each interview. Interviews were audio recorded with participant consent and lasted ∼40 to 60 min. AT used flexible probing throughout the interviews to encourage participants to elaborate on issues they considered important and to explore emerging topics in greater depth [29]. Field notes were maintained to document contextual observations, interview dynamics, and preliminary analytical reflections. All interviews were transcribed verbatim in Bangla and subsequently translated into English by AT, a native Bangla speaker with professional proficiency in English. After each interview, AT reviewed the findings and tracked their alignment with the research questions and objectives.

Data collection concluded after 12 interviews. This decision was informed by the principle of information power [31], which suggests that the sample size required in qualitative research depends on factors such as the specificity of the study aim, the richness and relevance of information provided by participants, and the analytical approach employed. In this study, participants occupied senior policy, program, and technical roles within Bangladesh’s nutrition sector and were selected for their direct experience with nutrition policy and implementation. Given the focused research objectives and the use of reflexive thematic analysis, the interviews generated sufficient depth and richness of information to address the study’s aims. Data collection concluded when successive interviews yielded no substantively new insights. Nevertheless, the relatively small sample size should be considered when interpreting the findings and is discussed further in the “study limitations” section.

### Data analysis

Data were analyzed using reflexive thematic analysis following the approach described by Braun and Clarke [32]. Analysis proceeded inductively, allowing themes to be generated from the data. The process followed 6 phases: familiarization with the data through repeated reading of transcripts; generation of initial codes; development of themes; review and refinement of themes; definition and naming of themes; and production of the final analytic narrative.

The integrated HPT–CDC conceptual framework served as an interpretive lens during the analysis. Although themes were generated inductively from participants’ accounts, the framework guided their interpretation with respect to the policy context, actors, content, and process. This approach allowed themes to emerge from participants’ accounts while ensuring that findings were systematically connected to the established health policy framework. The first author (AT) conducted the coding, whereas the senior author (HS) reviewed the coding framework and thematic interpretations to ensure consistency and credibility. Emerging themes were discussed iteratively with all coauthors to refine interpretations and strengthen the trustworthiness of the analysis. The themes were mapped onto the HPT–CDC domains. All analyses were performed using NVivo 15 (QSR International Pty Ltd., 2023).

## Results

### Overview of study participants

A total of 12 participants were included, representing 4 stakeholder groups (Table 1): international NGOs working on nutrition (*n* = 4), national research and academic institutions (*n* = 3), government nutrition authorities (*n* = 3), and subnational health and nutrition service providers (*n* = 2). Ten participants worked primarily at the national level in policy formulation, technical advisory, and program coordination roles, whereas 2 provided subnational perspectives from district-level implementation contexts.

**TABLE 1:** Study participant distribution by stakeholder group and engagement level

Stakeholder group	Number of participants	Level of engagement
International NGOs (including 2 United Nations agency representatives)	4	National
National Research and Academic Institutions	3	National
Government Nutrition Authorities	3	National
Subnational Health and Nutrition Services	2	Subnational

Abbreviation: NGO, nongovernmental organization.

### Thematic structure

The analysis generated 4 major themes that capture participants’ accounts of Bangladesh’s policy response to DBM (Table 2). Theme 1 examines how DBM is understood and framed at the policy level. Theme 2 explores the current nutrition policy landscape and its responsiveness to DBM. Theme 3 identifies implementation challenges constraining effective policy response. Theme 4 captures stakeholder perspectives on policy strengthening and future directions. Table 2 maps each theme and its subthemes to the corresponding dimensions of the integrated HPT–CDC conceptual framework, illustrating how identified themes connect to the framework’s domains and CDC policy stages.

**TABLE 2:** Thematic structure mapped to the integrated HPT–CDC framework

Theme	Subthemes	Framework dimension(s)	CDC policy stage(s)
Theme 1: understanding and framing DBM	1.1 Shifting the meaning of malnutrition from undernutrition to dual burden 1.2 Ongoing challenges in defining and conceptualizing DBM	• Context (nutrition transition, historical focus) • Actors (expert vs. community understanding)	• Problem identification
Theme 2: current nutrition policy landscape and responsiveness to DBM	2.1 Comprehensive multisectoral nutrition policies with a persistent focus on undernutrition 2.2 Absence of integrated DBM-specific frameworks and double-duty action approaches	• Content (policy substance) • Actors (multisectoral engagement) • Context (institutional arrangements)	• Policy analysis • Strategy and policy development
Theme 3: policy implementation challenges	3.1 Weak multisectoral coordination and policy implementation gaps 3.2 Weak monitoring, evaluation, and accountability systems 3.3 Insufficient human and institutional capacity for implementation	• Process (implementation mechanisms) • Actors (capacity, coordination) • Context (resource environment)	• Policy implementation • Evaluation
Theme 4: policy strengthening and future directions	4.1 Enhancing multisectoral governance, food system transformation, and regulatory interventions 4.2 Strengthening implementation capacity, monitoring systems, and institutional mechanisms 4.3 Behavior change communication and awareness programs	• All dimensions (comprehensive strengthening) • Context (governance reform) • Actors (capacity building) • Content (policy revision) • Process (implementation systems)	• Strategy and policy development • Stakeholder engagement and education • All stages (iterative improvement)

Abbreviations: CDC, Centers for Disease Control and Prevention; DBM, double burden of malnutrition; HPT, Health Policy Triangle.

### Theme 1: understanding and framing DBM

#### Subtheme 1.1: shifting the meaning of malnutrition from undernutrition to dual burden

Participants consistently described a fundamental shift in how malnutrition is conceptualized in Bangladesh, moving from a historically dominant focus on undernutrition to a growing recognition of a dual burden encompassing both undernutrition and overnutrition. This shift was linked to broader socioeconomic changes, including improvements in food security, reductions in poverty, and rising purchasing power, which have altered dietary patterns and nutritional risk profiles. As 1 government nutrition official explained:

“As food security has improved, poverty has declined, and purchasing power has increased, undernutrition has gradually fallen. However, this progress has been accompanied by a rise in overnutrition due to shifts in dietary patterns” (P1, Government nutrition authority).

Several participants noted that malnutrition was previously understood almost exclusively as undernutrition, with limited recognition of overnutrition as a public health concern. Reflecting this evolving perspective, another government official stated:

“Previously, our general understanding of malnutrition was limited to undernutrition only. However, both undernutrition and overnutrition fall under the category of malnutrition” (P7, Government nutrition authority).

Participants further emphasized that this transition is evident at both population and household levels. They described declines in stunting and undernutrition alongside a rapid increase in overweight and obesity, particularly in urban areas (P12; Supplementary Table 1). Participants also highlighted that the dual burden of malnutrition is increasingly observed both within and across households. Within households, undernutrition and overnutrition may coexist simultaneously, for example, where an overweight mother and an undernourished child live together. Across households in the same community, food-secure households were reported to experience overweight, whereas economically disadvantaged neighboring households continued to face undernutrition (P9; Supplementary Table 1).

Despite growing recognition of the dual burden among policy-level and technical actors, participants identified persistent gaps in community-level understanding. A senior researcher highlighted the disconnect between expert discourse and public awareness:

“The concept of double burden of malnutrition does not actually exist in Bangladesh. It is neither recognised nor understood at the individual or household level” (P4, National research institution).

#### Subtheme 1.2: ongoing challenges in defining and conceptualizing DBM

Participants reported persistent conceptual ambiguities that complicate problem identification and policy formulation for DBM in Bangladesh. The absence of a universally accepted definition creates uncertainty among policy actors about what constitutes DBM and what should be prioritized in interventions. As 1 government official explained:

“To be honest, there is still no globally accepted definition. There is only a working definition provided by WHO, which we generally follow” (P7, Government nutrition authority).

Participants also attributed definitional ambiguity to both historical context and evolving scientific understanding. Malnutrition discourse in Bangladesh has long been dominated by undernutrition, dating back to a time when overnutrition was viewed as a problem confined to Western countries. This historical framing creates enduring path dependencies continuing to shape how DBM is defined and understood (P3; Supplementary Table 2).

Although participants acknowledged that DBM understanding has expanded, they emphasized that prevailing definitions remain limited in scope. An international NGO representative highlighted that micronutrient deficiencies are often excluded from the conceptualization of DBM:

“By double burden of malnutrition, we generally mean 2 things: overnutrition and undernutrition. One is malnutrition and the other is overnutrition—that is what we understand. But along with this double burden, there is another thing that is very important, and that is micronutrient deficiency” (P8, International NGO representative).

Participants also reported persistent uncertainty regarding DBM conceptualization across age groups and life stages, highlighting the importance of a life-course perspective, noting that individuals may experience underweight and overweight at different life stages (P7; Supplementary Table 2).

The 2 subthemes above indicate persistent challenges in DBM understanding and framing. Recognition remains largely limited to expert levels, with limited community awareness. Conceptual ambiguities, including roles of micronutrient deficiencies and life-course considerations, leave policy actors working with incomplete frameworks. Historical undernutrition-focused policies further constrain the adoption of a dual-burden perspective. These problem-identification gaps extend throughout the policy process, contributing to content gaps and implementation challenges described in themes 2 and 3.

### Theme 2: current nutrition policy landscape and responsiveness to DBM

#### Subtheme 2.1: comprehensive multisectoral nutrition policies with persistent focus on undernutrition

Bangladesh’s NNP architecture formally acknowledges both malnutrition forms but with markedly different emphasis: undernutrition receives considerably more attention and detailed intervention guidance, whereas overnutrition is addressed only through generic behavior change communication and without programmatic specificity. Stakeholders confirmed this structural pattern and, critically, offered explanatory accounts of why it has persisted despite growing epidemiologic awareness of overnutrition.

Participants observed that although DBM is increasingly recognized in principle, policy guidance remains uneven in depth and specificity. As 1 government official explained:

“In the process of formulating the new policy, there is an initiative to address DBM, although the work has not yet begun” (P6, Government nutrition authority).

Despite comprehensive multisectoral frameworks, participants reported that historical undernutrition focus continues to shape policy priorities and implementation. Undernutrition dominates across ministries, influencing program design and operational practices. As 1 government official summarized:

“Unfortunately, everything is still undernutrition-centric. Whether we talk about programs or policies, the main focus remains undernutrition” (P7, Government nutrition authority).

Although policies acknowledge rising overweight and obesity, interventions, training, technical guidance, and communication materials remain largely undernutrition-focused, leaving frontline workers less equipped to respond to overnutrition across population groups (P2, P7, P8; Supplementary Table 3).

#### Subtheme 2.2: absence of integrated DBM-specific frameworks and double-duty action approaches

Participants reported that Bangladesh lacks a formal, integrated policy framework specifically designed to address DBM. This gap was attributed not only to structural limitations but also to prevailing mindsets among policymakers. A government nutrition official reflected on this evolving policy awareness, stating:

“The main reason it has not been included in the policy is that our mindset has not yet fully evolved. I have been emphasising this for the past two to three years. Our discourse has focused heavily on stunting—an issue we have been addressing for the last 20 years” (P7, Government nutrition authority).

Participants described this as a lag between epidemiological reality and policy cognition. Representatives from international NGOs and the government noted:

“Effective interventions, links, or strong separate policies for overnutrition are not there. That part is missing” (P8, International NGO representative).

“If you go to programmatic level… whether there is anything that specifically addresses the double burden of malnutrition with a double duty action approach, the answer is no” (P7, Government nutrition authority).

Additional perspectives from other participants further corroborated the absence of overnutrition-specific policy mechanisms within the existing national nutrition framework (Supplementary Table 4).

### Theme 3: policy implementation challenges

#### Subtheme 3.1: weak multisectoral coordination and policy implementation gaps

Participants identified a critical disconnect between formal structures and functional operationalization. Although comprehensive frameworks exist with explicit coordination mandates, on-the-ground translation was described as weak. An international NGO representative noted:

“Bangladesh performs well in policy adoption, comparable to many countries. But when it comes to implementation, we face significant challenges” (P9, international NGO representative).

This assessment was echoed across stakeholder groups. A UN agency representative and academic institution participants emphasized that although policies are well-developed, implementation falls short of the original intent, with implementation challenges rather than policy design identified as the primary constraint (P2, P5; Supplementary Table 5). Nutrition is inherently multisectoral, yet sectors operate largely in silos with limited functional coordination despite formal structures. As a UN agency representative explained:

“Nutrition is a multisectoral issue, not the responsibility of a single ministry. The Bangladesh National Nutrition Council (BNNC) under the Ministry of Health is supposed to coordinate across 22 ministries, but it lacks the mechanisms to regularly monitor or review progress” (P2, UN agency representative).

Weak coordination challenges extend beyond organizational structures to accountability gaps. Participants noted the absence of effective mechanisms to ensure that nonhealth sectors fulfill their nutrition-related responsibilities, limiting accountability and cross-sectoral commitment to nutrition objectives (P8; Supplementary Table 5).

#### Subtheme 3.2: weak monitoring, evaluation, and accountability systems

Despite formal provisions, participants identified persistent weaknesses in the functioning of monitoring, evaluation, and accountability mechanisms. Although monitoring policies formally exist, they lack rigorous mechanisms for effective oversight. As a government nutrition authority explained:

“The monitoring mechanism is very weak” (P6, Government nutrition authority).

Government monitoring lacks adequate strength because routine activity tracking fails to capture service quality or verify information accuracy provided to beneficiaries (P8; Supplementary Table 6). Participants reported gaps between formal monitoring systems outlined in policies and their practical application. A researcher highlighted this deficit:

“There is a monitoring policy, but in practice, it is not fully implemented” (P3, National research institution).

Participants identified critical gaps related to routine data collection and systematic evaluation. Key nutrition indicators, particularly anthropometric measurements, are not consistently tracked across service delivery points (P1, P3; Supplementary Table 6). Systematic weaknesses in evaluation compound this data gap, with agencies often lacking basic information on policy outcomes and unable to demonstrate implementation progress or impact (P10; Supplementary Table 6).

#### Subtheme 3.3: insufficient human and institutional capacity for implementation

Interviewed participants identified critical shortages of trained, dedicated personnel as major constraints affecting policy implementation and scale-up. Workforce limitations extend from national coordination bodies to frontline service delivery. At the national level, institutional capacity constraints are particularly evident in coordinating bodies. Despite formal mandates, BNNC was described as underresourced and lacking authority. A UN agency representative explained:

“BNNC is expected to coordinate across 22 ministries, but it has limited manpower and authority. Even though the government has given them a mandate, a small and weak directorate-level institution cannot effectively supervise so many ministries” (P2, UN agency representative).

Capacity constraints within technical institutions compound these challenges. Institutions like Institute of Public Health Nutrition (IPHN) lack the manpower required even for limited geographical implementation (P6; Supplementary Table 7). These institutional capacity deficits restrict the system’s ability to provide oversight, technical guidance, and quality assurance for nutrition programming.

At the frontline level, participants described substantial human resource gaps limiting nutrition service delivery. No specific nutrition workers exist at the field level, and family planning workers already carrying multiple priorities leave nutrition competing for attention (P8; Supplementary Table 7). Nutrition activities are often carried out by workers whose primary responsibility is not nutrition. A government nutrition authority explained:

“The primary problem is human resources. Nutrition has no dedicated human resources and depends on proxies who do not consider it their main task” (P7, Government nutrition authority).

Beyond workforce numbers, available personnel often lack the skills and training needed to implement nutrition interventions effectively. This skills gap reflects inadequate training, leaving workers unable to operationalize policies, even when they are explained. A UN agency representative highlighted:

“They have not been trained... even if you explain the policy, they cannot implement it because they are unaware” (P10, UN agency representative).

### Theme 4: policy strengthening and future directions

#### Subtheme 4.1: enhancing multisectoral governance, food system transformation, and regulatory interventions

Participants highlighted the need to strengthen multisectoral governance mechanisms and to expand policy responses beyond the health sector. Current coordination bodies face persistent institutional weaknesses, particularly regarding the capacity to fulfill coordination mandates effectively (P11; Supplementary Table 8). Stronger governance requires clearer role definitions, improved accountability, and broader stakeholder engagement. As an international NGO representative stated:

“It must clearly state the responsibilities of each stakeholder… otherwise there will be a big gap” (P8, International NGO representative).

Participants also highlighted the need to engage private-sector actors in nutrition governance as a critical strategy addressing emerging nutrition challenges (P10; Supplementary Table 8).

Stakeholders recognized healthy diets as a central component of efforts to address DBM and emphasized the need to move beyond a traditional focus on food security toward improving dietary quality. As an international NGO representative noted:

“Food security alone is not enough; attention must be given to food quality” (P9, International NGO representative).

A prominent regulatory concern raised was the sale of unregulated, unhealthy food near schools. Participants described widespread availability of highly processed, energy-dense snacks within school vicinities, emphasizing the need for enforceable restrictions. As 1 subnational health and nutrition service provider noted:

“In front of every school… there's always a fast-food shop. If they could be fined or restricted, it would help” (P11, Subnational health and nutrition services).

#### Subtheme 4.2: strengthening implementation capacity, monitoring systems, and institutional mechanisms

Building on implementation and monitoring gaps identified in theme 3, participants identified strategies enhancing Bangladesh’s ability to operationalize nutrition policies and respond effectively to DBM. Strengthening implementation was repeatedly highlighted as a priority, with participants noting that policies are strong but lack proper follow-through (P2; Supplementary Table 9). Systematic project assessment is essential for learning and improvement. As a district health education officer stated:

“If a three-month pilot is done but not evaluated, nothing improves” (P11, Subnational health and nutrition services).

Strengthening monitoring and verification systems is essential for accountability. Participants identified the need for functional surveillance systems enabling evidence-based decision-making. Proper record-keeping allows early problem identification and rapid response (P8; Supplementary Table 9).

Building human and institutional capacity emerged as a key priority to directly address workforce constraints. Frontline workers often lack specific skills in implementing nutrition policies effectively (P10; Supplementary Table 9). Strengthening coordination structures and ensuring adequate local-level resources were also recommended:

“The District Nutrition Coordination Committee needs to be made functional with proper support” (P11, Subnational health and nutrition services).

Participants highlighted emerging positive developments, such as research partnerships and digitized reporting systems that have the potential to be scaled up. Research on trans-fat was reported to have already contributed to the establishment of new national laboratory capacity (P9; Supplementary Table 9).

#### Subtheme 4.3: behavior change communication and awareness programs

Behavioral change communication (BCC) is widely recognized as a fundamental strategy for addressing both undernutrition and overnutrition in Bangladesh. Interviewed participants emphasized the central role of systematic communication and knowledge dissemination across stakeholder groups. A government nutrition official noted:

“The main strategy is counselling and communication aimed at changing behaviour” (P1, Government nutrition authority).

Participants highlighted mass media campaigns as key mechanisms for reaching wide audiences, drawing lessons from successful initiatives, such as oral rehydration solution awareness programs (P2; Supplementary Table 10). This example demonstrated how consistent public education translates into widespread knowledge and behavior change at the community level, providing a potential model for DBM-related nutrition messaging.

Health professionals play a critical role in initiating and sustaining BCC, even in the absence of formal policies or programs (P10; Supplementary Table 10). The absence of comprehensive overnutrition policies limits the development of structured BCC programs. A senior researcher explained:

“First, we don't have a comprehensive policy on overweight and obesity. Without it, awareness campaigns and training cannot be properly implemented” (P3, National research institution).

## Discussion

This study identified a range of conceptual, policy, and implementation challenges that stakeholders perceived as constraining effective responses to DBM in Bangladesh. Findings suggest that limitations in how DBM is understood, prioritized, and translated into policy and practice continue to hinder effective responses. Stakeholders highlighted challenges related to conceptual clarity, competing policy priorities, multisectoral coordination, and implementation capacity, which may impede the translation of growing awareness of DBM into coherent and sustained action. Addressing these challenges will require stronger alignment between policy objectives and implementation realities, as well as greater attention to how DBM is incorporated into policy planning, program design, and service delivery. Drawing on the perspectives of 12 senior nutrition stakeholders, this study contributes empirical evidence on perceived gaps in current policy responses and identifies stakeholder priorities for strengthening future efforts to address DBM in Bangladesh.

Themes 1 and 2 help explain why Bangladesh’s nutrition policies remain undernutrition-centric despite growing recognition of overnutrition. Stakeholders described a persistent “path dependency” [33], in which priorities established during decades of undernutrition programming continue to shape policy cognition, funding flows, and institutional culture. In this “adding on” dynamic, overnutrition concerns are appended to existing frameworks without meaningful reorientation, reflecting a lag between epidemiological reality and policy understanding [33,34]. The lack of a widely operationalized DBM definition further compounds this challenge, with ongoing ambiguity around what constitutes DBM, which age groups should be prioritized, and how micronutrient deficiencies fit within the dual burden framework. These conceptual gaps help explain the structural policy limitations identified in theme 2. Overnutrition remains insufficiently integrated into policy, highlighting that the absence of double-duty actions stems from institutional and cognitive factors that require engagement at the actor level, rather than simple policy revision [5].

Findings from theme 3 highlight implementation gaps as a central challenge in Bangladesh’s nutrition policy, reflecting a broader disconnect between policy intentions and practical realities in global nutrition governance [35,36]. Three interrelated barriers were identified: weak multisectoral coordination, insufficient monitoring and accountability systems, and persistent resource and workforce constraints. Although formal structures such as the BNNC exist, stakeholders described multisectoral coordination as largely symbolic, with nutrition often viewed by nonhealth sectors as outside their remit. This underscores the absence of a truly operational multisectoral approach, which is essential for effectively addressing DBM [37]. Coordination challenges were also apparent at subnational levels, in which platforms experienced irregular meetings and limited logistical support, indicating that gaps extend beyond national structures to the frontline settings in which interventions are delivered. These findings align with prior evidence that effective multisectoral nutrition action requires functional coordination at district and community levels, supported by adequate resources, clear mandates, and robust accountability frameworks [35,37,38].

Monitoring and accountability gaps were identified as a major constraint in subtheme 3.2. Although policy documents outline monitoring mechanisms, participants reported that their implementation remains weak. Existing data systems do not adequately capture service quality or provide timely information to guide decision-making. In particular, the lack of routine anthropometric measurements and the absence of DBM-specific indicators were highlighted as critical shortcomings. The 2020 Global Nutrition Report notes that when monitoring systems focus primarily on undernutrition while neglecting overweight, obesity, and diet-quality indicators, implementation tends to default to traditional undernutrition-focused interventions [10]. Accountability for nutrition outcomes is also limited, with few consequences for poor performance, reducing incentives for implementers to maintain high-quality service delivery [10,39,40].

Subtheme 3.3 highlighted human resource constraints as one of the most critical barriers to effective policy implementation. Participants described the absence of a dedicated nutrition workforce at the frontline, with existing staff already overburdened and nutrition responsibilities often treated as secondary tasks. Capacity is further constrained by a lack of DBM-specific skills, as most personnel are trained primarily in undernutrition-focused approaches. These findings align with previous evidence showing that inadequate workforce capacity undermines the effectiveness of nutrition programs across LMICs [[41], [42], [43], [44]]. Limited budget allocations for nutrition-specific interventions further exacerbate the challenge, indicating that fiscal commitments have not kept pace with policy rhetoric on DBM [10,45].

Reflecting the reform priorities highlighted in theme 4, participants identified several key areas for strengthening nutrition policy, including enhancing multisectoral governance, advancing food system reforms through regulatory measures, improving implementation capacity and monitoring, and expanding BCC initiatives. They emphasized the importance of functional, rather than symbolic, coordination mechanisms, echoing previous evidence that successful nutrition action requires clearly defined roles, robust accountability structures, and active engagement across sectors beyond health [46]. The call to strengthen subnational coordination is consistent with experiences from the “Nutrition Officers in Tanzania” initiative, in which effective implementation relied on empowered district-level platforms and clearly defined authority [47].

Emphasis on food system approaches—including trans-fat regulation, sodium reduction, nutrient profiling, and restrictions on unhealthy foods near schools—reflects alignment with global evidence on obesity prevention [48,49]. However, participants noted that although such regulations exist, enforcement remains weak, highlighting the need for implementation infrastructure, including laboratory capacity, inspection systems, and clear enforcement authority. Expanding the definition of food safety to encompass nutritional harm represents an important conceptual shift, with the potential to strengthen regulatory action [49,50].

The emphasis on implementation capacity and monitoring reflects recognition that Bangladesh’s primary challenge lies in execution rather than policy formulation. Participants stressed the importance of evaluation and learning, noting that “if a pilot is done but not evaluated, nothing improves,” underscoring the role of learning systems in adapting policies. Emerging research partnerships and digitized reporting systems provide a foundation for continuous quality improvement, but these mechanisms require further scaling and institutionalization. Participants also highlighted BCC as a critical component of effective nutrition policy because it shapes dietary knowledge, attitudes, and practices that influence nutritional outcomes. However, they noted that BCC efforts in Bangladesh remain heavily focused on undernutrition, with comparatively limited and less developed initiatives targeting overnutrition and unhealthy diets [8,51,52].

Taken together, the 4 themes of this study highlight that a DBM-responsive policy architecture is not simply a matter of appending overnutrition programs to existing undernutrition frameworks. Instead, it requires what Hawkes et al. [5] describe as “double-duty actions”: interventions, programs, and policies deliberately designed or adapted to address the shared drivers of undernutrition, overweight, obesity, and diet-related noncommunicable diseases within the same populations or households [5]. The *Lancet* series on DBM further identifies such actions with strong evidence potential, categorized around 4 key domains: early-life nutrition, diet diversity, food environments, and socioeconomic factors [5].

Bangladesh has existing platforms that could be adapted for double-duty purposes. Breastfeeding promotion already features in the NNP [23] and NPAN-2; enhancing enforcement of the International Code of Marketing of Breast-milk Substitutes and expanding community lactation support could strengthen both undernutrition and obesity prevention [5]. The National School Feeding Policy [53,54] could be adapted to emphasize dietary diversity, locally sourced foods, and limits on added sugar, salt, and saturated fat, simultaneously addressing micronutrient deficiencies and unhealthy dietary exposure [55,56]. Social protection programs such as the Mother and Child Benefit Programme can act as double-duty interventions if paired with BCC focusing on diet quality [57]. Finally, food environment interventions such as front-of-pack labeling [58,59] and marketing restrictions on unhealthy foods would reduce exposure to ultraprocessed foods, benefiting both undernourished and overweight populations [53]. These examples highlight that Bangladesh does not need to develop a DBM policy from scratch; rather, existing programs can be systematically adapted, monitored, and integrated through a double-duty lens, using combined indicators to capture co-benefits and manage potential trade-offs.

### Implications for policy and practice

Our findings have several important implications for nutrition policy in Bangladesh and similar contexts. First, although existing programs provide opportunities for double-duty interventions, addressing DBM ultimately requires moving beyond “policy add-ons” toward a broader reorientation of nutrition governance, including integrated policy frameworks, stronger multisectoral coordination, and balanced resource allocation. Second, strengthening implementation must be prioritized alongside policy formulation, with particular emphasis on improving multisectoral coordination, establishing functional monitoring and accountability systems, and building adequate workforce capacity. Third, conceptual clarity around DBM definitions and operationalization is essential for translating recognition into action. Fourth, greater emphasis is needed on food system approaches, including regulatory measures and environmental modifications, to complement individual-level interventions.

For policymakers, ongoing revisions such as NPAN-3 provide an opportunity to integrate DBM into national frameworks, clarify implementation processes, strengthen monitoring indicators for both forms of malnutrition, and align resource allocation with current nutrition trends. For program implementers, the findings highlight the need for workforce training on integrated approaches, strengthened supervision systems, and adaptation of intervention packages to address risk factors associated with both forms of malnutrition.

### Study Limitations

This study has several limitations that should be considered when interpreting the findings. First, although DBM is inherently a multisectoral issue and participants frequently emphasized the importance of engagement beyond the nutrition sector, all participants were recruited from nutrition-sector organizations. Consequently, perspectives from stakeholders in other sectors relevant to DBM, such as agriculture, education, food systems, trade, and urban planning, were not captured. Future research could extend this work by examining how actors across these sectors understand and respond to DBM. Second, the sample size was relatively small. Although participants were purposively selected for their direct involvement in nutrition policy and implementation, and the sample size was guided by the principle of information power, the findings may not reflect the full range of perspectives present within Bangladesh’s broader nutrition and policy landscape. Relatedly, data collection was conducted over a 3-mo period (June–August 2025), which limited the time available for more participants’ recruitment. A longer recruitment period may have facilitated engagement with additional senior stakeholders, particularly from academic and research institutions.

Third, the cross-sectional design captured stakeholder perspectives at a single point in time during ongoing policy review and implementation processes. As nutrition policies, governance arrangements, and programmatic priorities continue to evolve, stakeholder perspectives may also change over time. Longitudinal research could provide further insights into how understandings of DBM and policy responses develop in response to evolving policy contexts. Fourth, transcripts were not returned to participants for comment or correction, and member checking of emergent themes was not conducted because of time and scheduling constraints that limited the feasibility of a second round of stakeholder consultation. Finally, participant responses may have been influenced by social desirability bias, particularly when discussing politically sensitive issues such as policy implementation challenges, institutional constraints, and resource allocation. Efforts were made to minimize this risk by assuring participants of confidentiality and encouraging open discussion during interviews.

## Conclusions

Drawing on the perspectives of 12 senior stakeholders within Bangladesh’s nutrition sector, this study provides timely, stakeholder-informed evidence to inform nutrition governance during a critical policy revision period. We recommend 3 priority actions for policymakers: *1*) operationalize DBM definitions within NPAN-3 with clear indicators and resource allocations; *2*) establish effective multisectoral coordination mechanisms with dedicated budgets and robust accountability systems at both national and subnational levels; and *3*) invest in workforce capacity to support integrated approaches that address both undernutrition and overnutrition. Development partners can reinforce these efforts by aligning technical assistance with double-duty action frameworks, improving monitoring systems that capture the full spectrum of malnutrition, and facilitating evidence exchange with countries navigating similar transitions. It is evident that recognizing DBM in policy is necessary but insufficient without deeper reforms in governance, implementation, and resource allocation—an emerging challenge for many LMICs undergoing rapid nutrition transitions.

## Author contributions

The authors’ contributions were as follows – AT, HS: conceptualized and designed the study. AT: conducted the key informant interviews (KII), collected the data, led the data analysis, and drafted the manuscript; AT, SKC: contributed to the interpretation of the results; HS, MK, DG: supervised the analytical process; HS, MK, SKC, DG: critically revised the manuscript for intellectual content; and all authors: reviewed and edited subsequent drafts, read and approved the final version, and contributed to the decision to submit the manuscript for publication.

## Ethical considerations

Ethical approval was obtained from the Human Research Ethics Committee, Australian National University, Australia (Project H/2025/0278) and the Research and Innovation Centre, Khulna University, Bangladesh (KUECC-2025-04-32). All participants provided oral informed consent and were informed of their right to withdraw at any time. To ensure confidentiality, participants were assigned alphanumeric codes (P1–P12). Audio recordings and identifying information were stored separately in password-protected files accessible only to the first author. Data will be retained for 5 y and then securely destroyed in accordance with institutional guidelines.

## Declaration of Generative AI and AI-assisted technologies in the writing process

The author(s) declare that no generative AI or AI-assisted technologies were used in the writing of this manuscript.

## Data availability

The data are available from the corresponding author upon reasonable request.

## Funding

The authors reported no funding received for this study.

## Conflict of interest

The authors report no conflicts of interest.
