# Supplementary material for: The Double Burden of Malnutrition in Bangladesh: Policy Architecture, Implementation Realities, and Future Directions
Source: Curr Dev Nutr. 2026 Jul 25;10(8):109433. doi: 10.1016/j.cdnut.2026.109433 (PMC13542226; doi:10.1016/j.cdnut.2026.109433)
Supplement: Supplementary file 1 [file mmc1.docx]

**Supplementary Tables**

**Title: The Double Burden of Malnutrition in Bangladesh: Policy Architecture, Implementation Realities, and Future Directions**

**1st Author: Ashis Talukder**

| **Supplementary Table 1: Additional quotes supporting Theme 1, Sub-theme 1.1** | |
| --- | --- |
| **Participant** | **Quote** |
| **P12, Subnational health and nutrition services** | *“Previously, undernutrition and stunting were highly prevalent, but those have decreased markedly. At the same time, overnutrition or obesity in urban areas has increased significantly. I would say obesity has increased more than undernutrition”* |
| **P9, International NGO representative** | *“Both forms of malnutrition, undernutrition and overnutrition, can coexist simultaneously within the same household”* |

| **Supplementary Table 2: Additional quotes supporting Theme 1, Sub-theme 1.2** | |
| --- | --- |
| **Participant** | **Quote** |
| **P3, National research institution** | *“Earlier, when we started our careers back in the late 1990s, our understanding of malnutrition in Bangladesh and in low- and middle-income countries was mainly about undernutrition: a child becoming stunted or wasted, with overnutrition either ignored or considered a problem confined to Western countries”* |
| **P7, Government nutrition authority** | *“We must ask which age group we are referring to… we should consider the individual-level phenomenon: the same person may experience both problems at different life stages—underweight at one stage and becoming overweight later”* |

| **Supplementary Table 3: Additional quotes supporting Theme 2, Sub-theme 2.1** | |
| --- | --- |
| **Participant** | **Quote** |
| **P9, International NGO representative** | *“Operational planning documents, particularly NPAN-2, were described as adopting multisectoral framing by addressing undernutrition, overnutrition, anemia, and related health concerns”* |
| **P9, International NGO representative** | *“This policy was described as emphasizing food quality, dietary diversity, and the structural determinants of malnutrition affecting both rural and urban households”* |
| **P1, Government nutrition authority** | *“Earlier in our country, malnutrition was understood almost entirely as undernutrition, and our primary focus was on addressing that”* |
| **P2, UN agency representative** | *“The National Nutrition Policy strongly covers undernutrition, while overnutrition is included but not prominently”* |
| **P8, International NGO representative** | *“For underweight mothers, what message to give is there, but for overweight, no one really knows what message to give”* |
| **P7, Government nutrition authority** | *“All existing training, orientation, and skill development initiatives have been undernutrition centric… all communication materials, orientation programs, and trainings focus exclusively on undernutrition”* |

| **Supplementary Table 4: Additional quotes supporting Theme 2, Sub-theme 2.2** | |
| --- | --- |
| **Participant** | **Quote** |
| **P6, Government nutrition authority** | *“Although undernutrition policies have also covered overnutrition, there is no extensive framework on it”* |

| **Supplementary Table 5: Additional quotes supporting Theme 3, Sub-theme 3.1** | |
| --- | --- |
| **Participant** | **Quote** |
| **P5, Academic institution** | *“The main challenge remains implementation, which is not progressing as effectively as policy design”* |
| **P8, International NGO representative** | *“Since nutrition is multisectoral, we must keep in mind whether other sectors are acting responsibly”* |
| **P2, UN agency representative** | *“Implementation remains a major gap. Bangladesh performs well in policy adoption, comparable to many countries. But when it comes to implementation, we face significant challenges”* |

| **Supplementary Table 6: Additional quotes supporting Theme 3, Sub-theme 3.2** | |
| --- | --- |
| **Participant** | **Quote** |
| **P8, International NGO representative** | *“The government does some monitoring… they do not fully follow accountability”* |
| **P1, Government nutrition authority** | *“Anthropometric measurements—such as height and weight—are not routinely collected”* |
| **P3, National research institution** | *“What struck me most is that anthropometric measurements for malnutrition patients, which need to be done regularly, are often not carried out”* |
| **P10, UN agency representative** | *“If you ask them whether they have data showing how this policy has been implemented or its impact over the last two years to five years, they do not know”* |

| **Supplementary Table 7: Additional quotes supporting Theme 3, Sub-theme 3.3** | |
| --- | --- |
| **Participant** | **Quote** |
| **P8, International NGO representative** | *“Beyond community clinics, there is very little communication at the grassroots level. There are no specific nutrition workers at the field level, and family planning workers already have so many priorities that nutrition often falls behind”* |
| **P6, Government nutrition authority** | *“The Institute of Public Health Nutrition is supposed to carry out this work. But even if it tried to implement activities in just one area of Dhaka, it does not have the manpower required”* |

| **Supplementary Table 8: Additional quotes supporting Theme 4, Sub-theme 4.1** | |
| --- | --- |
| **Participant** | **Quote** |
| **P11, Subnational health and nutrition services** | *“Meetings are supposed to be held every two months, but these meetings don’t happen… they lack the logistical support or resources needed”* |
| **P10, UN agency representative** | *“If private sector actors do not focus on health or nutrition, nutrition outcomes will be compromised”* |
| **P5, Academic institution** | *“The National Food and Nutrition Security Policy (2020) were developed along with CIP-3…. a healthy diet as a key strategy to tackle both under- and overnutrition”* |
| **P5, Academic institution** | *“We are developing a Nutrient Profile Model and sodium benchmarks to regulate unhealthy diets”* |

| **Supplementary Table 9: Additional quotes supporting Theme 4, Sub-theme 4.2** | |
| --- | --- |
| **Participant** | **Quote** |
| **P2, UN agency representative** | *“Implementation needs to be our main focus. Policies are strong, but without proper follow-through, we cannot expect improvements”* |
| **P8, International NGO representative** | *“If proper record-keeping exists, then we can identify problems early and act quickly”* |
| **P10, UN agency representative** | *“They have not been trained… even if you explain the policy, they cannot implement it because they are unaware”* |
| **P9, International NGO representative** | *“Research on trans-fat has already led to new national laboratory capacity”* |

| **Supplementary Table 10: Additional quotes supporting Theme 4, Sub-theme 4.3** | |
| --- | --- |
| **Participant** | **Quote** |
| **P2, UN agency representative** | *“Now you can find ORS even in small grocery stores, and people generally know how much to take, when to take it”* |
| **P10, UN agency representative** | *“We must not just sit and wait. From our side, we should also actively raise public awareness about which factors cause obesity and overweight”* |
