# Supplementary material for: The Double Burden of Malnutrition in Bangladesh: Policy Architecture, Implementation Realities, and Future Directions
Source: Curr Dev Nutr. 2026 Jul 25;10(8):109433. doi: 10.1016/j.cdnut.2026.109433 (PMC13542226; doi:10.1016/j.cdnut.2026.109433)
Supplement: Supplementary file 2 [file mmc2.docx]

**Supplementary File 1**

**Key Informant Interview Guide**

**Title: The Double Burden of Malnutrition in Bangladesh: Policy Architecture, Implementation Realities, and Future Directions**

# Study Overview

This interview guide was developed to explore the perspectives of senior-level nutrition stakeholders on the double burden of malnutrition (DBM) and related policy responses in Bangladesh. The guide was informed by a review of relevant literature and structured around the integrated Health Policy Triangle–CDC Policy Process Framework (HPT–CDC), which examines the context, actors, content, and process dimensions of nutrition policy.

The guide is organised into four thematic domains corresponding to the study objectives: (1) awareness and understanding of DBM; (2) assessment of current national nutrition policy architecture; (3) multisectoral coordination, stakeholder roles, and implementation experiences; and (4) future policy directions and priorities. Open-ended questions are used throughout to encourage participants to introduce issues beyond those explicitly listed.

This guide was used for semi-structured key informant interviews (KIIs) conducted face-to-face interviews.

# Instructions for the Interviewer

**Before beginning each interview, please ensure the following:**

- Confirm that informed consent has been obtained and the participant understands their right to withdraw at any time.
- Remind the participant that the interview will be audio-recorded (with consent) and that all information will be kept confidential.
- Assign the participant an alphanumeric code (P1–P12) and record it on the interview log.
- Note the date, time, location, and participant category in the field notes.

**During the interview:**

- Use the questions as a flexible guide, not a rigid script. Follow the participant's lead where relevant new issues emerge.
- Use probing questions (listed in italics below each main question) to encourage depth and elaboration.
- Avoid leading questions; allow silences and avoid interrupting participants mid-response.
- Record field notes on contextual observations, interview dynamics, and preliminary analytical reflections immediately after each interview.

# Opening and Introduction

Thank you for agreeing to participate in this study. My name is **Ashis Talukder**, and I am a researcher at the Australian National University conducting a study on nutrition policy in Bangladesh, with a particular focus on the double burden of malnutrition. The interview will take approximately 40–60 minutes. There are no right or wrong answers—I am interested in your experiences and perspectives.

With your permission, I will audio-record this interview to ensure accuracy. Your name will not be used in any publication; you will be referred to by a code. You are free to skip any question or stop the interview at any time.

Do you have any questions before we begin? [Obtain verbal consent and commence recording.]

# Domain 1: Awareness and Understanding of the Double Burden of Malnutrition

This section explores how participants understand and frame the concept of DBM in the context of Bangladesh's nutrition transition.

**1.1.** In your view, how would you describe the current nutrition situation in Bangladesh? How has it changed over the past decade or so?

- What types of malnutrition are you most concerned about in your work?
- Have you observed any changes in the prevalence of overweight or obesity alongside undernutrition?

**1.2.** How do you understand the term 'double burden of malnutrition'? Could you explain what it means in the Bangladeshi context?

- Do you think there is a shared or consistent understanding of DBM among nutrition stakeholders in Bangladesh?
- Are there aspects of DBM—such as micronutrient deficiencies or life-course considerations—that you feel are not adequately captured in current definitions?

**1.3.** To what extent do you think the coexistence of undernutrition and overnutrition is recognised at different levels among policymakers, programme staff, and communities?

- Have you noticed differences in awareness between national, subnational, and community levels?
- What do you think are the main reasons for any gaps in recognition or understanding?

**1.4.** How do you think Bangladesh's broader development changes, such as urbanisation, economic growth, and dietary shifts, have influenced the nutritional landscape?

- Have you observed differences between rural and urban areas, or between different socioeconomic groups?
- To what extent do you think food system changes have contributed to rising overnutrition?

# Domain 2: National Nutrition Policy Architecture and Responsiveness to DBM

This section explores stakeholders' perceptions of existing national nutrition policies and their adequacy in addressing DBM.

**2.1.** How would you assess the current national nutrition policy architecture in Bangladesh—for example, the National Nutrition Policy (2015), NPAN-2, and related frameworks?

- In your view, how well do these policies address undernutrition? Overnutrition? Both simultaneously?
- Are there specific areas or population groups that you feel are not adequately addressed?

**2.2.** Do you think current policies adequately reflect the double burden of malnutrition as an integrated challenge, or do they tend to address undernutrition and overnutrition separately?

- Can you give examples of policies or programmes that you see as responsive—or not responsive—to DBM?
- Is there a policy or framework you are aware of that specifically addresses DBM in an integrated way?

**2.3.** Are you familiar with the concept of 'double-duty actions'—interventions designed to address both undernutrition and overnutrition simultaneously? If so, how do you think they are reflected in current policy or programming?

- Can you identify any existing programmes in Bangladesh that could serve as double-duty actions if adapted?
- What do you think would be needed to incorporate double-duty approaches into the policy framework?

**2.4.** What factors do you think explain why policy responses to overnutrition remain less developed than those targeting undernutrition in Bangladesh?

- Do you think institutional culture, cognitive factors, or historical priorities play a role?
- How do you think funding flows and donor priorities influence the focus of nutrition policies?

**2.5.** How do you see the National Food and Nutrition Security Policy (2020) and its Plan of Action (2021–2030) contributing to addressing DBM? Are there gaps?

- Does the food systems approach embedded in this policy provide opportunities to address both forms of malnutrition?
- Are overnutrition-related targets—such as limiting overweight prevalence—reflected in programme planning and monitoring?

# Domain 3: Stakeholder Roles, Coordination, Implementation, and Accountability

This section examines stakeholders' experiences of multisectoral coordination, implementation challenges, and accountability mechanisms in nutrition policy.

**3.1.** What is your organisation's role in nutrition policy development and/or programme implementation in Bangladesh?

- How does your organisation engage with DBM-related issues in its day-to-day work?
- Have your organisation's priorities shifted in recent years in response to rising overnutrition?

**3.2.** How would you describe the current mechanisms for multisectoral coordination in nutrition, such as the Bangladesh National Nutrition Council (BNNC)?

- In your experience, how functional are these coordination platforms in practice?
- What are the main strengths and weaknesses of existing coordination mechanisms?
- Are there sectors beyond health, such as agriculture, education, or trade, that you think should be more actively engaged in nutrition governance?

**3.3.** In your experience, what are the most significant barriers to translating nutrition policies into effective implementation at the national level? At the subnational or frontline level?

- How do resource constraints—including budgetary and human resources—affect implementation?
- Are there differences in implementation capacity between national and subnational levels?
- What role does political will or institutional leadership play in enabling or constraining implementation?

**3.4.** How would you assess existing monitoring and evaluation systems for nutrition programmes in Bangladesh?

- Are there specific indicators used to track DBM outcomes—covering both undernutrition and overnutrition?
- How effectively is monitoring data used to inform policy and programme decision-making?
- What accountability mechanisms exist to ensure that stakeholders fulfil their nutrition-related responsibilities?

**3.5.** How would you characterise the current workforce capacity to implement nutrition interventions, particularly at the district and community levels?

- Are frontline workers adequately trained to respond to both undernutrition and overnutrition?
- What skills or training do you think are most lacking in the current nutrition workforce?
- Are there dedicated nutrition staff at the field level, or is nutrition integrated into other roles?

**3.6.** In your view, how effectively do stakeholders from different sectors and organisations communicate and collaborate on nutrition issues?

- Are there examples of coordination that has worked well? What made it successful?
- What have been the main obstacles to effective inter-agency or multisectoral collaboration?

# Domain 4: Future Policy Directions and Priorities

This section explores stakeholders' recommendations for strengthening nutrition policy to address DBM more effectively.

**4.1.** In your opinion, what are the most urgent policy priorities to address the double burden of malnutrition in Bangladesh?

- Are there specific regulatory or legislative measures you would prioritise, for example, food labelling, marketing restrictions, or food safety standards?
- How should existing programmes be adapted to become more responsive to DBM?

**4.2.** With NPAN-3 currently in development, what recommendations would you make to ensure it more effectively addresses DBM compared to its predecessors?

- What specific targets, indicators, or accountability mechanisms would you recommend including?
- How should the balance between undernutrition and overnutrition be reflected in NPAN-3?
- What role should civil society, academic institutions, and the private sector play in the development and implementation of NPAN-3?

**4.3.** How do you think multisectoral governance for nutrition can be strengthened in Bangladesh?

- What changes to existing coordination structures, such as BNNC, would make them more functional?
- How can accountability for nutrition outcomes be strengthened across sectors?
- Are there models from other countries or sectors that Bangladesh could learn from?

**4.4.** What strategies do you think are most important for improving workforce capacity to address DBM at both national and subnational levels?

- What training or capacity-building initiatives would you recommend?
- How can nutrition roles and responsibilities be better integrated into existing health and social service systems?

**4.5.** What role do you think behaviour change communication (BCC) should play in addressing DBM in Bangladesh? How does current BCC compare to what is needed?

- How can BCC strategies be expanded beyond undernutrition to include overnutrition and unhealthy dietary practices?
- What channels or approaches do you think would be most effective for reaching different population groups?

**4.6.** Are there any policy responses or governance models from other countries experiencing similar nutrition transitions that you think Bangladesh could usefully adapt?

- What aspects of the international experience do you think are most relevant to Bangladesh's context?

**4.7.** Is there anything else you would like to add about the double burden of malnutrition, existing policies, or future directions that we have not yet covered?

# Closing

Thank you very much for your time and for sharing your perspectives. Your insights are very valuable to this research. As a reminder, all information you have shared will be kept strictly confidential and used only for research purposes. You will be identified only by an alphanumeric code in any publications arising from this study.

If you think of anything further after this interview, or have any questions about the study, please feel free to contact the research team using the contact details provided in your participant information sheet.

[Stop audio recording. Document field notes. Complete interview log.]

# Supplementary Information

## Participant Categories

Participants were recruited from four stakeholder groups:

- Government nutrition authorities (national and subnational)
- International non-governmental organisations working on nutrition
- National research and academic institutions
- Subnational health and nutrition service providers

## Conceptual Framework Alignment

Interview domains were aligned with the integrated Health Policy Triangle–CDC Policy Process Framework (HPT–CDC) as follows:

| **Interview Domain** | **HPT Dimension** | **CDC Policy Stage(s)** |
| --- | --- | --- |
| Domain 1: DBM Awareness and Understanding | Context; Actors | Problem Identification |
| Domain 2: National Policy Architecture | Policy Content; Context | Policy Analysis; Strategy & Policy Development |
| Domain 3: Coordination, Implementation, Accountability | Actors; Process | Policy Enactment; Policy Implementation |
| Domain 4: Future Policy Directions | All dimensions | Evaluation; Stakeholder Engagement |

## Ethical Note

All interviews were conducted after obtaining informed oral consent. Participants were assured of confidentiality and assigned alphanumeric codes in all records. Audio recordings and identifying information were stored separately in password-protected files accessible only to the first author.

Ethical approval was obtained from the Human Research Ethics Committee, Australian National University, Australia (Project H/2025/0278), and the Research and Innovation Centre, Khulna University, Bangladesh (KUECC-2025-04-32).

**End of Interview Guide**
