# Supplementary material for: The Double Burden of Malnutrition in Bangladesh: Policy Architecture, Implementation Realities, and Future Directions
Source: Curr Dev Nutr. 2026 Jul 25;10(8):109433. doi: 10.1016/j.cdnut.2026.109433 (PMC13542226; doi:10.1016/j.cdnut.2026.109433)
Supplement: Supplementary file 3 [file mmc3.docx]

**Supplementary File 2**

**Consolidated Criteria for Reporting Qualitative Research (COREQ)**

**32-Item Checklist for Interviews and Focus Groups**

**Title: The Double Burden of Malnutrition in Bangladesh: Policy Architecture, Implementation Realities, and Future Directions**

| **No.** | **Item** | **Guide Question** | **Response from This Study** |
| --- | --- | --- | --- |
| **Domain 1: Research Team and Reflexivity** | | | |
| ***Personal Characteristics*** | | | |
| **1** | **Interviewer/facilitator** | Which author/s conducted the interview or focus group? | The first author (A.T.) conducted all 12 key informant interviews face-to-face. |
| **2** | **Credentials** | What were the researcher's credentials, e.g., PhD, MD? | A.T. is a PhD candidate at the Australian National University, National Centre for Epidemiology and Population Health. H.S. (senior author) holds a PhD and has extensive experience in qualitative health policy research in LMICs. |
| **3** | **Occupation** | What was their occupation at the time of the study? | A.T. was a PhD candidate conducting this study as part of doctoral research. H.S. was an academic researcher and supervisor at the Australian National University. |
| **4** | **Gender** | Was the researcher male or female? | Male |
| **5** | **Experience and training** | What experience or training did the researcher have? | A.T. had prior experience conducting research on nutrition and health-related topics in Bangladesh. Before data collection, A.T. received training and mentorship in qualitative interviewing techniques—including probing, active listening, reflexive note-taking, and management of interviewer bias—from H.S. H.S. provided ongoing methodological oversight throughout the study. After the first two interviews, H.S. reviewed transcripts and field notes and provided structured feedback, which informed minor refinements to the interviewing approach. |
| ***Relationship with Participants*** | | | |
| **6** | **Relationship established** | Was a relationship established prior to study commencement? | No. A.T. was not affiliated with any of the participating institutions and had no prior professional, supervisory, or working relationships with the participants. |
| **7** | **Participant knowledge of the interviewer** | What did participants know about the researcher, e.g., personal goals, reasons for doing the research? | Participants were provided with study information before consenting, including the study aims and the institutional affiliation of the research team (Australian National University). They were informed that A.T. was conducting the study as part of doctoral research on nutrition policy in Bangladesh. |
| **8** | **Interviewer characteristics** | What characteristics were reported about the interviewer/facilitator? | A.T. is a Bangladeshi national, a native Bangla speaker with professional proficiency in English, and was independent of all participating organisations. This position minimised potential influence from organisational relationships, professional hierarchies, or conflicts of interest on participant responses. |
| **Domain 2: Study Design** | | | |
| ***Theoretical Framework*** | | | |
| **9** | **Methodological orientation and theory** | What methodological orientation was stated to underpin the study, e.g., grounded theory, discourse analysis, ethnography, phenomenology, content analysis? | The study employed a qualitative design using semi-structured key informant interviews (KIIs). Data were analysed using reflexive thematic analysis following Braun and Clarke (2006), with themes generated inductively from participants' accounts. The integrated Health Policy Triangle–CDC Policy Process Framework (HPT–CDC) served as an interpretive lens to contextualise and organise emergent themes within established health policy frameworks. |
| ***Participant Selection*** | | | |
| **10** | **Sampling** | How were participants selected, e.g., purposive, convenience, consecutive, snowball? | Participants were selected using purposive sampling to obtain a range of perspectives from stakeholders involved in nutrition policy and programming in Bangladesh. Initial participants were identified through a review of nutrition policy documents, national coordination meeting records, and relevant literature. Additional participants were recruited through snowball sampling. |
| **11** | **Method of approach** | How were participants approached, e.g., face-to-face, telephone, mail, email? | Potential participants were contacted by email and provided with study information before providing informed consent. |
| **12** | **Sample size** | How many participants were in the study? | 12 participants. |
| **13** | **Non-participation** | How many people refused to participate or dropped out? Reasons? | Exact numbers of individuals who were approached but declined to participate or did not respond were not formally tracked, given the purposively selected pool of stakeholders and reliance on snowball referrals. All individuals who agreed to participate completed interviews; no participant withdrew after consenting. |
| ***Setting*** | | | |
| **14** | **Setting of data collection** | Where was the data collected, e.g., home, clinic, workplace? | Face-to-face interviews were conducted in Bangladesh between June and August 2025. The specific interview locations (e.g., participants' workplaces) are not described in detail in the manuscript. |
| **15** | **Presence of non-participants** | Was anyone else present besides the participants and researchers? | No other individuals were present during the interviews aside from the participant and the interviewer (A.T.). |
| **16** | **Description of sample** | What are the important characteristics of the sample, e.g., demographic data, date? | 12 senior-level nutrition stakeholders participated, representing four groups: international NGOs working on nutrition (n=4), national research and academic institutions (n=3), government nutrition authorities (n=3), and subnational health and nutrition service providers (n=2). Ten participants worked at the national level in policy formulation, technical advisory, and programme coordination roles; two provided subnational perspectives from district-level implementation contexts. All participants were Bangladeshi nationals based in Dhaka and fluent in Bangla. |
| ***Data Collection*** | | | |
| **17** | **Interview guide** | Were questions, prompts, guides provided by the authors? Was it pilot tested? | A semi-structured interview guide was developed by A.T. following a review of relevant literature and informed by the integrated HPT–CDC conceptual framework. It was refined through iterative discussions within the research team to ensure conceptual alignment with study objectives, clarity of wording, and logical sequencing of questions. The final version is provided in Supplementary File 1. |
| **18** | **Repeat interviews** | Were repeat interviews carried out? If yes, how many? | No repeat interviews were conducted. Each participant was interviewed once. |
| **19** | **Audio/visual recording** | Did the research use audio or visual recording to collect the data? | Yes. All interviews were audio-recorded with participant consent. |
| **20** | **Field notes** | Were field notes made during and/or after the interview or focus group? | Yes. Field notes were maintained to document contextual observations, interview dynamics, and preliminary analytical reflections after each interview. |
| **21** | **Duration** | What was the duration of the interviews or focus group? | Approximately 40–60 minutes per interview. |
| **22** | **Data saturation** | Was data saturation discussed? | Data collection was guided by the principle of information power rather than saturation per se. Collection concluded after 12 interviews, when successive interviews yielded no substantively new insights, given the focused research objectives, seniority of participants, and use of reflexive thematic analysis. |
| **23** | **Transcripts returned** | Were transcripts returned to participants for comment and/or correction? | Transcripts were not returned to participants for comment or correction, due to time and scheduling constraints that limited the feasibility of a second round of stakeholder consultation. This is acknowledged as a limitation of the study (see 'Study Limitations'). |
| **Domain 3: Analysis and Findings** | | | |
| ***Data Analysis*** | | | |
| **24** | **Number of data coders** | How many data coders coded the data? | The first author (A.T.) conducted the coding, while the senior author (H.S.) reviewed the coding framework and thematic interpretations to ensure consistency and credibility. Emerging themes were discussed iteratively with all co-authors to refine interpretations and strengthen the trustworthiness of the analysis. |
| **25** | **Description of the coding tree** | Did authors provide a description of the coding tree? | Yes. Four major themes and associated sub-themes are described in detail in the Results section, and Table 2 maps each theme and sub-theme to the corresponding HPT–CDC framework dimensions and CDC policy stages. |
| **26** | **Derivation of themes** | Were themes identified in advance or derived from the data? | Themes were derived inductively from the data. The HPT–CDC conceptual framework served as an interpretive lens during analysis but did not predetermine themes; it guided the interpretation of inductively generated themes with respect to policy context, actors, content, and process. |
| **27** | **Software** | What software, if applicable, was used to manage the data? | NVivo 15 (QSR International Pty Ltd., 2023) was used for data management and analysis. |
| **28** | **Participant checking** | Did participants provide feedback on the findings? | Participants did not provide feedback on the emergent themes or findings, due to the same time and scheduling constraints noted in item 23. This is also acknowledged as a limitation of the study (see 'Study Limitations'). |
| ***Reporting*** | | | |
| **29** | **Quotations presented** | Were participant quotations presented to illustrate the themes/findings? Was each quotation identified, e.g., participant number? | Yes. Representative verbatim quotations are presented throughout the Results section to illustrate each theme and sub-theme. Each quotation is attributed to a participant code (e.g., P1–P12) and stakeholder category (e.g., Government nutrition authority, International NGO representative). |
| **30** | **Data and findings consistent** | Was there consistency between the data presented and the findings? | Yes. Findings are supported by direct quotations from participants and cross-referenced with supplementary tables (Supplementary Tables 1–10) containing additional supporting excerpts. The thematic structure maps systematically to the interview domains and the HPT–CDC framework. |
| **31** | **Clarity of major themes** | Were major themes clearly presented in the findings? | Yes. Four major themes with nine sub-themes are presented, each with a descriptive heading, explanatory narrative, supporting participant quotations, and cross-references to the HPT–CDC framework dimensions and CDC policy stages (Table 2). |
| **32** | **Clarity of minor themes** | Is there a description of diverse cases or discussion of minor themes? | Divergent and less prominent perspectives are noted within themes where relevant (e.g., variation between national and subnational levels, differences in stakeholder group perspectives). The limitations section acknowledges that the relatively small sample may not reflect the full range of perspectives in Bangladesh's nutrition sector. |

**Note: KII = Key Informant Interview; HPT-CDC = Health Policy Triangle-CDC Policy Process Framework; DBM = Double Burden of Malnutrition; NVivo = qualitative data analysis software. NA: Not Available/Not Reported**

**Ref: Tong A, Sainsbury P, Craig J. Consolidated criteria for reporting qualitative research (COREQ): a 32-item checklist for interviews and focus groups. Int J Qual Health Care. 2007 Dec;19(6):349-57. doi: 10.1093/intqhc/mzm042. Epub 2007 Sep 14. PMID: 17872937.**
